# Serum Anti-oligodendrocyte Autoantibodies in Patients With Multiple Sclerosis Detected by a Tissue-Based Immunofluorescence Assay

**DOI:** 10.3389/fneur.2021.681980

**Published:** 2021-08-05

**Authors:** Yukino Miyachi, Takayuki Fujii, Ryo Yamasaki, Daisuke Tsuchimoto, Kyoko Iinuma, Ayako Sakoda, Shoko Fukumoto, Takuya Matsushita, Katsuhisa Masaki, Noriko Isobe, Yusaku Nakabeppu, Jun-ichi Kira

**Affiliations:** ^1^Department of Neurology, Neurological Institute, Graduate School of Medical Sciences, Kyushu University, Fukuoka, Japan; ^2^Department of Immunobiology and Neuroscience, Medical Institute of Bioregulation, Kyushu University, Fukuoka, Japan; ^3^Translational Neuroscience Center, Graduate School of Medicine, School of Pharmacy at Fukuoka, International University of Health and Welfare, Fukuoka, Japan; ^4^Department of Neurology, Brain and Nerve Center, Fukuoka Central Hospital, International University of Health and Welfare, Fukuoka, Japan

**Keywords:** multiple sclerosis, oligodendrocyte, autoantibody, progression, disability

## Abstract

Multiple sclerosis (MS), the most prevalent inflammatory disease of the central nervous system (CNS), is characterized by damaged to myelin sheaths and oligodendrocytes. Because MS patients have variable clinical courses and disease severities, it is important to identify biomarkers that predict disease activity and severity. In this study, we assessed the frequencies of serum autoantibodies against mature oligodendrocytes in MS patients using a tissue-based immunofluorescence assay (IFA) to determine whether anti-oligodendrocyte antibodies are associated with the clinical features of MS patients and whether they might be a biomarker to assess CNS tissue damage in MS patients. We assessed the binding of serum autoantibodies to mouse oligodendrocytes expressing Nogo-A, a reliable mature oligodendrocyte marker, by IFA with mouse brain and sera from 147 MS patients, comprising 103 relapsing–remitting MS (RRMS), 22 secondary progressive MS (SPMS), and 22 primary progressive MS (PPMS) patients, 38 neuromyelitis optica spectrum disorder (NMOSD) patients, 23 other inflammatory neurological disorder (OIND) patients, and 39 healthy controls (HCs). Western blotting (WB) was performed using extracted mouse cerebellum proteins and IgG from anti-oligodendrocyte antibody-positive MS patients. Tissue-based IFA showed that anti-oligodendrocyte antibodies were positive in 3/22 (13.6%) PPMS and 1/22 (4.5%) SPMS patients but not in RRMS, NMOSD, and OIND patients or HCs. WB demonstrated the target CNS proteins recognized by serum anti-oligodendrocyte antibodies were approximately 110 kDa and/or 150 kDa. Compared with anti-oligodendrocyte antibody-negative MS patients, MS patients with anti-oligodendrocyte antibodies were significantly older at the time of serum sampling, scored significantly higher on the Expanded Disability Status Scale and the Multiple Sclerosis Severity Score, and had a higher frequency of mental disturbance. Although the clinical significance of anti-oligodendrocyte antibodies is still unclear because of their low frequency, anti-oligodendrocyte autoantibodies are potential biomarkers for monitoring the disease pathology and progression in MS.

## Introduction

Multiple sclerosis (MS) is an inflammatory demyelinating disease of the central nervous system (CNS). MS is caused by a complex interplay between B and T lymphocytes, glial cells (oligodendrocytes, microglia, and astrocytes), and neurons (1). Because MS patients show a variable clinical course, disease severity, and therapeutic response (2, 3), it is important to identify biomarkers that predict disease activity, disease severity, and response to treatment so as to guide neurologists to appropriate treatment decisions in MS. Ideal biomarkers should be safe and obtained by non-invasive or minimally invasive methods (4). In this regard, serum autoantibodies against CNS-specific antigens released during tissue destruction might be candidate biomarkers (5).

We previously reported serum CNS-specific antinuclear antibodies (ANA) in MS patients (6). CNS-specific ANA positivity was determined by indirect immunofluorescence assay (IFA) showing positive staining for mouse CNS nuclei (CNS-ANA-positive) and negative staining for laryngeal carcinoma-derived human epithelial type-2 cell nuclei (conventional ANA-negative). Serum CNS-specific ANA were significantly more frequent in MS patients than in neuromyelitis optica spectrum disorder (NMOSD) patients or in healthy controls (HCs) and were more common in secondary progressive MS (SPMS) than relapsing–remitting MS (RRMS) patients. Moreover, MS patients with CNS-specific ANA against the 55-kDa band showed a higher frequency of SPMS and cortical gray matter lesions, and higher Kurtzke Expanded Disability Status Scale (EDSS) scores (7) and Multiple Sclerosis Severity Scores (MSSS) (8) than those without CNS-specific ANA, which indicates that CNS-specific ANA might be a serum biomarker to assess CNS tissue damage in MS. These observations prompted us to investigate whether serum autoantibodies against CNS-resident glial cells might also be a biomarker that reflects CNS tissue damage in MS.

The most specific pathological changes in MS are well-demarcated focal lesions with primary demyelination whereby myelin sheaths and oligodendrocytes are destroyed and axons are partly preserved (9). Therefore, we focused on autoantibodies against oligodendrocytes in the sera of MS patients as a candidate biomarker that reflects CNS tissue damage and disease severity. In the present study, we assessed the prevalence of anti-oligodendrocyte antibodies in patients with RRMS, SPMS, primary progressive MS (PPMS), NMOSD, and other inflammatory neurological disorders (OINDs), as well as HCs, using IFA with mouse brain and examined their association with the clinical features of MS patients.

## Materials and Methods

### Subjects

One hundred and forty-seven MS patients, comprising 103 RRMS, 22 SPMS, and 22 PPMS patients, 38 NMOSD patients with anti-aquaporin 4 (AQP4)-IgG, 23 OIND patients with CNS lesions including 5 cases with neuro-Behçet's disease, 5 cases with cerebral infarction probably caused by cerebral vasculitis, 4 cases with CNS lupus, 4 cases with Sjögren's syndrome myelopathy, 3 cases with neurosarcoidosis, 2 cases with neuro-Sweet's disease, and 39 HCs with sufficient remaining sera for IFA collected from 2014 to 2018 at a single MS center in Kyushu University Hospital were enrolled in this study (Table 1). MS was diagnosed according to the 2010 McDonald criteria (10), and NMOSD patients were diagnosed according to the 2015 NMOSD criteria (11). MS patients who were in remission, negative for AQP4-IgG, and had not received corticosteroids for at least 6 months prior to serum sampling were enrolled. Disease-modifying drugs (DMDs) were used in 63 of 147 MS patients (42.9%): 46 of 103 RRMS patients (44.7%) received DMDs including interferon (IFN)β-1a, IFNβ-1b, glatiramer acetate, dimethyl fumarate, and fingolimod; 9 of 22 SPMS patients (40.9%) were treated with DMDs including IFNβ-1b, dimethyl fumarate, and fingolimod; and 8 of 22 PPMS patients (36.4%) were treated with DMDs including IFNβ-1a, dimethyl fumarate, and fingolimod. NMOSD patients who were in remission, positive for AQP4-IgG, and had not received prednisolone ≥ 15 mg/day or immunosuppressive agents for at least 6 months prior to the serum sampling were also enrolled.

**Table 1:** Demographics and anti-oligodendrocyte antibody status of the study participants.

	**PPMS patients(*n* = 22)**	**RRMS patients(*n* = 103)**	**SPMS patients(*n* = 22)**	**NMOSD patients(*n* = 38)**	**OIND patients(*n* = 23)**	**HCs(*n* = 39)**
Female, *n* (%)	11 (50.0)	79 (76.7)	16 (72.7)	29 (76.3)	13 (56.5)	25 (64.1)
Age at time of serum sampling (mean ± SD), years	50.6 ± 11.8	42.7 ± 12.9	53.7 ± 12.3	51.1 ± 15.4	46.8 ± 11.7	44.1 ± 13.0
Disease duration at time of serum sampling (mean ± SD), years	13.3 ± 6.6	12.4 ± 9.6	25.4 ± 15.4	11.3 ± 10.1	9.1 ± 8.9	NA
Patients with anti-oligodendrocyte autoantibodies *n* (%)	3 (13.6)	0 (0.0)	1 (4.5)	0 (0.0)	0 (0.0)	0 (0.0)

*HCs, healthy controls; MS, multiple sclerosis; NA, not applicable; NMOSD, neuromyelitis optica spectrum disorders; OIND, other inflammatory neurological disorder; PPMS, primary progressive multiple sclerosis; RRMS, relapsing–remitting multiple sclerosis; SD, standard deviation; SPMS, secondary progressive multiple sclerosis*.

### Clinical Measures

We collected the demographic and clinical data of the participants including sex, age at time of serum sampling, age at disease onset, disease duration at time of serum sampling, EDSS score at time of serum sampling, MSSS at time of serum sampling, MS subtype, use of DMDs, serum autoantibodies (ANA, anti-double-stranded DNA antibodies, anti-Sjögren's syndrome A and B antibodies, and anti-thyroid peroxidase antibodies), cerebrospinal fluid (CSF) cell counts, CSF protein, positivity for CSF oligoclonal IgG bands (OBs), abnormal IgG index, and affected CNS areas on magnetic resonance imaging by retrospective review of the medical records. The IgG index was considered elevated if it was >0.658 (12).

### Tissue-Based IFA

Adult male C57BL/6 mice (10–12 weeks old) were perfused with chilled 4% paraformaldehyde in phosphate-buffered saline. The cerebrum, cerebellum, spinal cord (SC), dorsal root ganglia (DRG), and dorsal nerve roots were removed, fixed in 10% buffered formalin, and processed into paraffin sections (4 μm thick). Sera from human subjects (1:50 dilution) were absorbed with mouse liver powder (10 mg in 300 μl) (Rockland, Gilbertsville, ME, USA) using a rotator for 60 min at 4°C, as described previously (13). Following centrifugation at 15,000 r/min for 10 min, the supernatant was applied to the paraffin sections at 37°C. After a 1-h incubation and washing, bound IgG was detected with Alexa Fluor 488-conjugated goat anti-human IgG antibodies (Thermo Fisher Scientific, Waltham, MA, USA, 1:1,000). Nuclei were counterstained with 4′,6-diamidino-2-phenylindole (Vector Laboratories, Burlingame, CA, USA). Tissues were observed with a BZ-X700 fluorescence microscope (Keyence, Tokyo, Japan).

### Anti-oligodendrocyte Autoantibodies

We screened serum anti-oligodendrocyte autoantibodies, which bound to oligodendrocytes located in the mouse cerebellum, using IFA with sera from MS, NMOSD, and OIND patients and HCs. An experienced neuropathologist in the field of demyelinating diseases of the CNS (K.M.) and two investigators (T.F. and Y.M.), all of whom were blind to the clinical information, identified anti-oligodendrocyte autoantibodies on IFA by consensus. We performed double immunostaining for patient IgG and anti-Nogo A antibodies (Santa Cruz Biotechnology, Dallas, TX, USA), a reliable mature oligodendrocyte marker (14), in the cerebrum, cerebellum, and SC to confirm the binding of patient IgG to mature oligodendrocytes. In addition, anti-myelin oligodendrocyte glycoprotein (MOG) antibodies (Sigma-Aldrich, St. Louis, MO, USA), a marker of myelin sheaths (14), and anti-S100β antibodies (Abcam, Cambridge, UK), a marker of Schwann cells in the peripheral nervous system (15), were also used as primary antibodies for double immunostaining with patient IgG. Species-specific Alexa Fluor 594-conjugated anti-IgG antibodies (Thermo Fisher Scientific, 1:1,000) were used as secondary antibodies.

### Western Blotting

The cerebellum and sciatic nerves were obtained from adult male C57BL/6 mice (10–12 weeks old) and homogenized in radio-immunoprecipitation assay buffer (Nacalai Tesque Inc., Kyoto, Japan). Homogenates were clarified by centrifugation at 14,000 × g for 10 min at 4°C, and supernatants were stored at −80°C. Extracted mouse cerebellum proteins were mixed with Laemmli sample buffer and heated at 98°C for 5 min. Proteins were separated by sodium dodecyl sulfate polyacrylamide gel electrophoresis (SDS-PAGE) and transferred onto polyvinylidene difluoride membranes (Millipore, Hertfordshire, UK). Membranes were blocked in 2% ECL Prime Blocking Reagent (GE Healthcare Life Sciences, New York, NY, USA), cut into strips, and incubated with sera from seropositive patients, seronegative patients, HCs (each at 1:300 dilution), or anti-Nogo A antibodies (Santa Cruz Biotechnology, 1:200 dilution) for 1 h at room temperature (RT), as described previously (16). Membranes were washed and incubated with the appropriate horseradish peroxidase-conjugated secondary antibodies (Southern Biotechnology Associates, Birmingham, AL, USA) for 1 h at RT. After washing, signals were detected using the ChemiDoc XRS system (Bio-Rad, Hercules, CA, USA).

### Immunoadsorption Assay

To identify the molecular weight (MW) of the target protein that anti-oligodendrocyte antibodies recognized, we prepared different MW fractions of mouse cerebellum. Extracted mouse cerebellum proteins were run through Amicon Ultra-4 Centrifugal Filter Devices (Millipore) including membranes with a nominal MW limit of 100, 50, and 30 kDa (17). By this method, extracts were fractionated to three fractions based on their MW. The fractions were collected as a residue (R): R100 (>100 kDa), R50 (100 kDa > R50 > 50 kDa), and R30 (50 kDa > R30 > 30 kDa). Next, we performed immunoadsorption tests with the three fractions (R100, R50, and R30) of mouse cerebellum by IFA. For IFA, sera from seropositive MS patients were diluted 1:50, incubated with one of the three fractions (R100, R50, or R30), whole extracted mouse cerebellum proteins as a positive control antigen, or bovine serum albumin (BSA) as a negative control antigen at the same concentration (600 μg/ml) for 1 h at 4°C, and used as the primary antibody (16). After the reaction, we performed IFA on mouse cerebellum as described above.

### Immunoprecipitation

Extracted mouse cerebellum proteins were precleared using FG bead-protein G (Tamagawa Seiki, Nagano, Japan). Total protein was mixed with patient IgG and incubated for 1 h at 4°C. Antigen–antibody immunocomplexes were subsequently subjected to IP with FG bead-protein G for 2 h at 4°C. The beads were washed with IP buffer. The proteins were eluted with SDS sample buffer and heated at 98°C for 5 min. IP samples were then separated using SDS-PAGE and visualized by silver staining.

### Ethics Statement

The research protocol for the retrospective study and the data privacy procedures for consented human samples were approved by the Kyushu University Ethics Committee (30-159 and 2019-203). Animal experiments were performed according to the protocols approved by the Institutional Animal Care and Use Committee at Kyushu University (A19-109).

### Statistical Analysis

All analyses were performed using JMP 14.0.0 software (SAS Institute Inc., Cary, NC, USA). The Mann–Whitney *U*-test was used to compare continuous variables, and the chi-square test or Fisher's exact probability test (when criteria for the chi-square test were not fulfilled) was used to compare categorical variables between two groups. Uncorrelated *p*-values (*p*^*uncorr*^) were corrected by multiplying them by the number of comparisons (Bonferroni–Dunn's correction) to calculate corrected *p*-values (*p*^*corr*^) (18). In all analyses, statistical significance was set at *p* < 0.05.

## Results

### Demographic Features of MS and NMOSD Patients and HCs

There were no significant differences in sex between all groups after a correction was made (Tables 1, 2). RRMS patients were significantly younger at the time of serum sampling than SPMS patients or NMOSD patients (mean ± SD; 42.7 ± 12.9 vs. 53.7 ± 12.7, *p*^*corr*^ = 0.0045; and vs. 51.1 ± 15.4, *p*^*corr*^ = 0.018, respectively). Moreover, SPMS patients had a significantly longer disease duration at the time of serum sampling compared with RRMS patients or OIND patients (mean ± SD, years; 25.4 ± 15.4 vs. 12.4 ± 9.6, *p*^*corr*^ < 0.0001; and vs. 9.1 ± 8.9, *p*^*corr*^ = 0.001).

**Table 2:** Comparison of demographic data and anti-oligodendrocyte antibody status of the study participants.

	**Comparison groups**	***p^***uncorr***^***	***p^***corr***^***
Female/male	PPMS vs. RRMS	0.0177	NS
	PPMS vs. SPMS	NS	NS
	PPMS vs. NMOSD	0.0493	NS
	PPMS vs. OIND	NS	NS
	PPMS vs. HC	NS	NS
	RRMS vs. SPMS	NS	NS
	RRMS vs. NMOSD	NS	NS
	RRMS vs. OIND	NS	NS
	RRMS vs. HC	NS	NS
	SPMS vs. NMOSD	NS	NS
	SPMS vs. OIND	NS	NS
	SPMS vs. HC	NS	NS
	NMOSD vs. OIND	NS	NS
	NMOSD vs. HC	NS	NS
	OIND vs. HC	NS	NS
Age at time of serum	PPMS vs. RRMS	0.011	NS
sampling	PPMS vs. SPMS	NS	NS
	PPMS vs. NMOSD	NS	NS
	PPMS vs. OIND	NS	NS
	PPMS vs. HC	NS	NS
	RRMS vs. SPMS	0.0003	0.0045
	RRMS vs. NMOSD	0.0012	0.018
	RRMS vs. OIND	NS	NS
	RRMS vs. HC	NS	NS
	SPMS vs. NMOSD	NS	NS
	SPMS vs. OIND	0.0299	NS
	SPMS vs. HC	0.0347	NS
	NMOSD vs. OIND	NS	NS
	NMOSD vs. HC	0.0218	NS
	OIND vs. HC	NS	NS
Disease duration at time of	PPMS vs. RRMS	NS	NS
serum sampling	PPMS vs. SPMS	0.0088	NS
	PPMS vs. NMOSD	NS	NS
	PPMS vs. OIND	0.0317	NS
	RRMS vs. SPMS	<0.0001	<0.0001
	RRMS vs. NMOSD	NS	NS
	RRMS vs. OIND	0.0474	NS
	SPMS vs. NMOSD	NS	NS
	SPMS vs. OIND	0.0001	0.001
	NMOSD vs. OIND	NS	NS
Patients with	PPMS vs. RRMS	0.0048	NS
anti-oligodendrocyte	PPMS vs. SPMS	NS	NS
autoantibodies	PPMS vs. NMOSD	0.045	NS
	PPMS vs. OIND	NS	NS
	PPMS vs. HC	0.0428	NS
	RRMS vs. SPMS	NS	NS
	RRMS vs. NMOSD	NS	NS
	RRMS vs. OIND	NS	NS
	RRMS vs. HC	NS	NS
	SPMS vs. NMOSD	NS	NS
	SPMS vs. OIND	NS	NS
	SPMS vs. HC	NS	NS
	NMOSD vs. OIND	NS	NS
	NMOSD vs. HC	NS	NS
	OIND vs. HC	NS	NS

*The Mann–Whitney U-test was used to compare continuous variables, and the chi-square test or Fisher's exact probability test (when criteria for the chi-square test were not fulfilled) was used to compare categorical variables between two groups. Uncorrelated p-values (p^uncorr^) were corrected by multiplying them by the number of comparisons (Bonferroni–Dunn's correction) to calculate corrected p-values (p^corr^). In all analyses, statistical significance was set at p < 0.05*.

*HCs, healthy controls; MS, multiple sclerosis; NMOSD, neuromyelitis optica spectrum disorders; NS, not significant; OIND, other inflammatory neurological disorder; PPMS, primary progressive multiple sclerosis; RRMS, relapsing–remitting multiple sclerosis; SPMS, secondary progressive multiple sclerosis; vs., versus*.

### Anti-oligodendrocyte Antibodies Detected by Tissue-Based IFA

**Figure 1:**
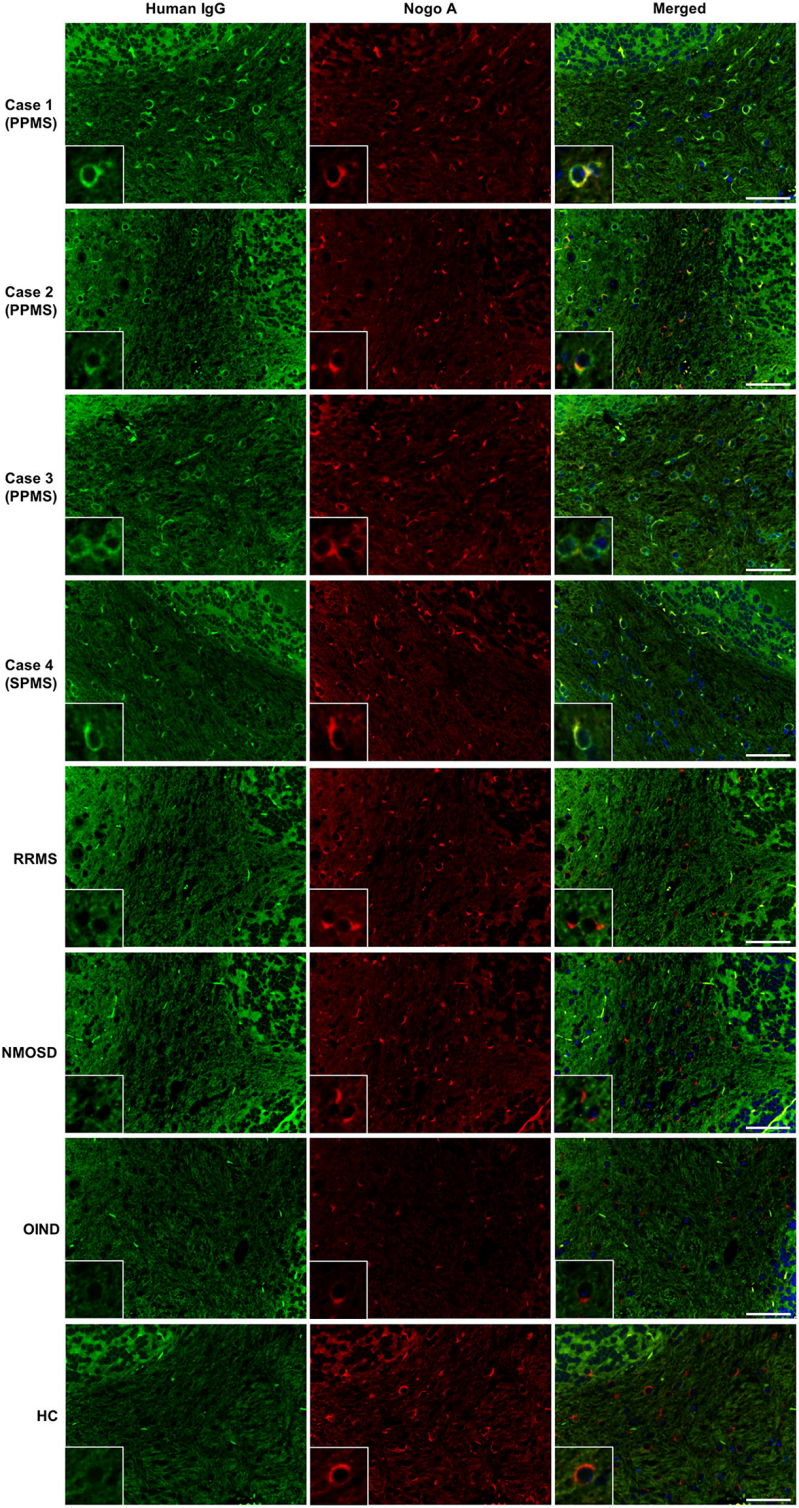
Screening for serum autoantibodies against oligodendrocytes in patients with MS. IgGs (green) from three PPMS patients (Cases 1–3) and one SPMS patient (Case 4) bound to Nogo A-positive oligodendrocytes (red) in mouse cerebellum, whereas control IgGs from one representative RRMS patient, one representative NMOSD patient, one representative OIND patient with neuro-Behçet's disease, and one representative HC showed no significant immunoreactivity toward Nogo A-positive oligodendrocytes. Nuclei were counterstained with 4′,6-diamidino-2-phenylindole (DAPI) (blue). Scale bars: 50 μm. ANA, antinuclear antibody; CNS, central nervous system; HC, healthy control; IgG, immunoglobulin G; MS, multiple sclerosis; NMOSD, neuromyelitis optica spectrum disorders; OIND, other inflammatory neurological disorder; PPMS, primary progressive multiple sclerosis; RRMS, relapsing–remitting multiple sclerosis; SPMS, secondary progressive multiple sclerosis.

**Figure 2:**
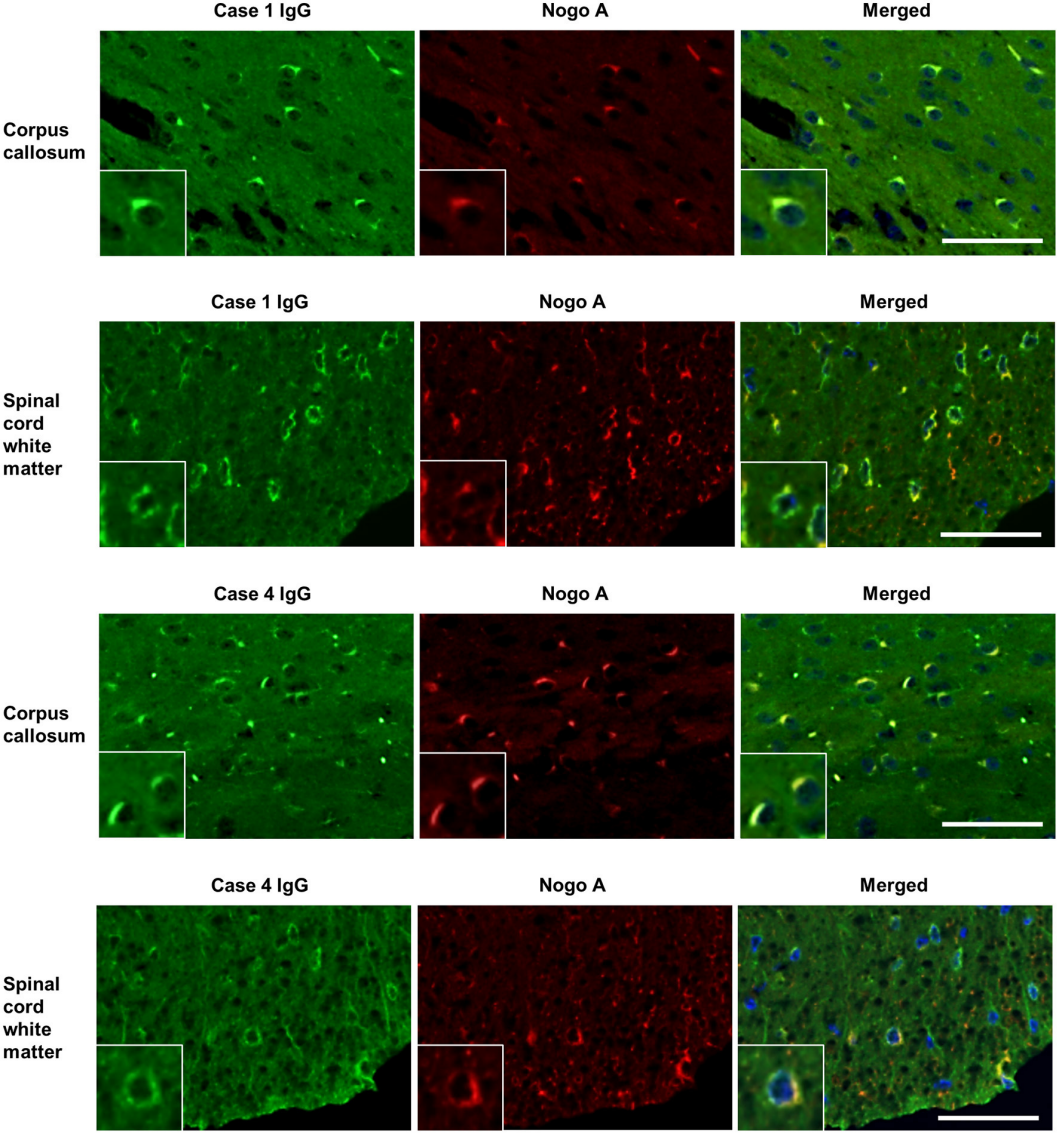
Dual immunostaining of mouse cerebrum and spinal cord using patient IgG and anti-Nogo A antibodies. IgGs (green) from one representative PPMS patient (Case 1) and one SPMS patient (Case 4) bound to Nogo A-positive oligodendrocytes (red) in mouse corpus callosum and spinal cord white matter. Scale bar = 50 μm. IgG, immunoglobulin G; PPMS, primary progressive multiple sclerosis; RRMS, relapsing–remitting multiple sclerosis.

**Figure 3:**
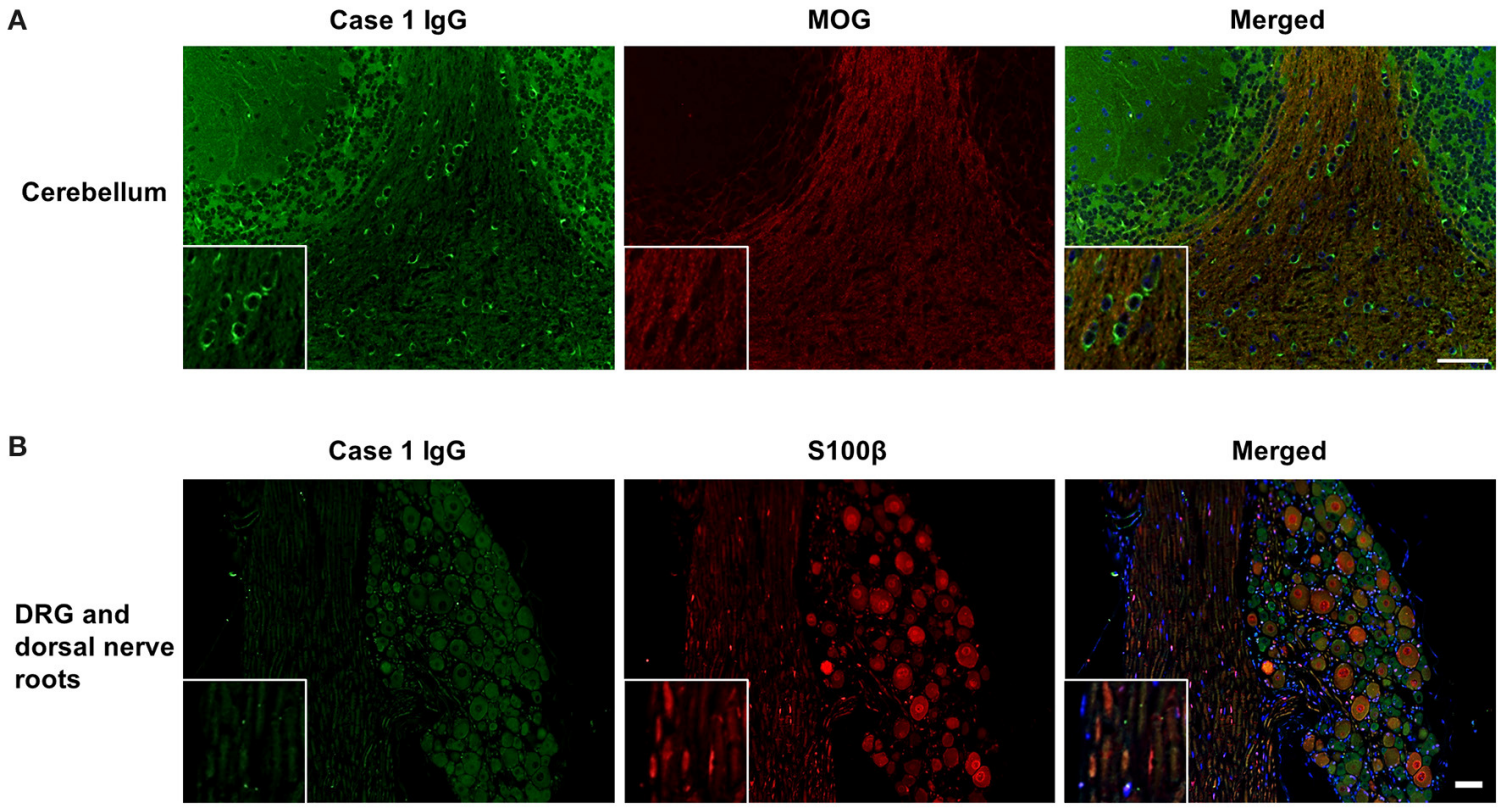
Dual immunostaining of mouse cerebellum using patient IgG and anti-MOG antibodies and of mouse DRG and dorsal nerve roots using anti-S100β antibodies**. (A)** Immunostaining of MOG (red), expressed in myelin sheaths but less frequently in the cytoplasm of oligodendrocytes, did not co-localize with IgG binding (green) from a representative MS patient with anti-oligodendrocyte antibodies (Case 1 in Figure 1). **(B)** IgG (green) from a representative MS patient with anti-oligodendrocyte antibodies (Case 1 in Figure 1) showed no significant immunoreactivity to mouse DRG neurons and dorsal nerve roots including S100β-positive Schwann cells (red) from the peripheral nervous system. Scale bar = 50 μm. DRG, dorsal root ganglion; IgG, immunoglobulin G; MS, multiple sclerosis; MOG, myelin oligodendrocyte glycoprotein.

IgG from all RRMS, NMOSD, and OIND patients and HCs showed no significant immunoreactivity to oligodendrocytes by IFA in the mouse cerebellum (Figure 1). In contrast, 3 of 22 PPMS patients (13.6%; Cases 1, 2, 3 in Figure 1) and 1 of 22 SPMS patients (4.5%; Case 4 in Figure 1) had serum IgG that bound to mouse Nogo A-labeled oligodendrocytes not only in the cerebellum but also in the corpus callosum and SC (Figures 1, 2). These autoantibodies bound to the cytoplasm of oligodendrocytes and did not co-localize with MOG, which is expressed in myelin sheaths but less frequently in the cytoplasm of oligodendrocytes (14) (Figure 3A). Moreover, these autoantibodies showed no significant immunoreactivity to mouse DRG neurons and dorsal nerve roots including S100β-positive Schwann cells (Figure 3B). The positivity rate of anti-oligodendrocyte antibodies was higher in PPMS patients than in RRMS patients (13.6% vs. 0.0%, *p*^*uncorr*^ = 0.0048), NMOSD patients (13.6% vs. 0.0%, *p*^*uncorr*^ = 0.045), or HCs (13.6% vs. 0.0%, *p*^*uncorr*^ = 0.0428) (Tables 1, 2), but all these differences lost statistical significance after a correction was made (Table 2).

### Immunochemical Characterization of Autoantigens

**Figure 4:**
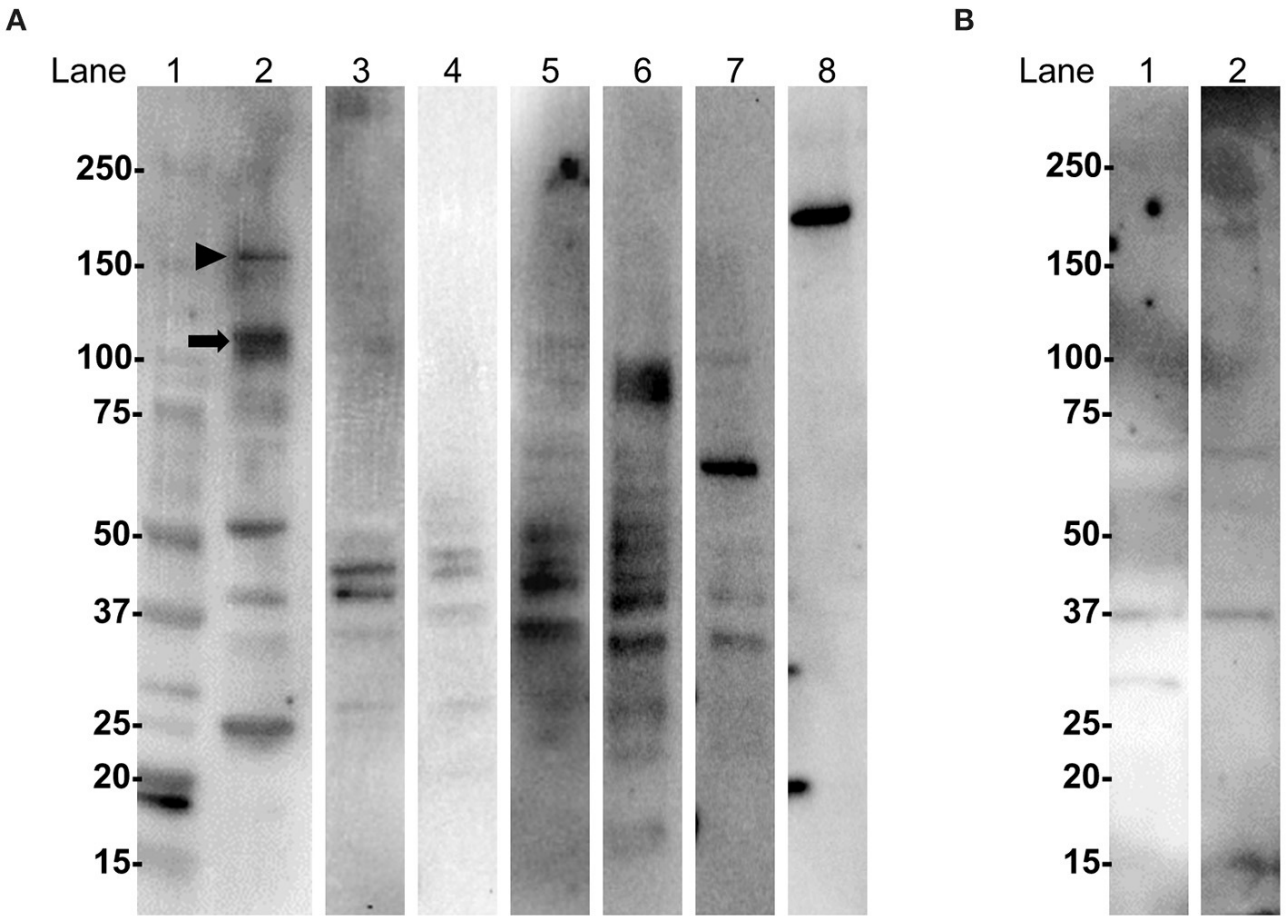
WB with IgG from MS patients and anti-oligodendrocyte antibodies using extracted mouse cerebellum and sciatic nerve proteins. **(A)** WB analysis of extracted mouse cerebellum. Lane 1: molecular weight markers; lanes 2, 3, 4, and 5: IgG from Cases 1, 2, 3, and 4 with anti-oligodendrocyte antibodies in Figure 1, respectively; lane 6: IgG from a RRMS patient without anti-oligodendrocyte antibodies; lane 7: IgG from a HC; lane 8: commercial anti-Nogo A antibody. IgG from Case 1 (lane 2) bound to proteins of approximately 110 kDa (arrow) and 150 kDa (arrowhead). IgG from Cases 2 (lane 3), 3 (lane 4), and 4 (lane 5); IgG from a RRMS patient without anti-oligodendrocyte antibodies (lane 6); or a HC (lane 7) did not bind to specific protein bands. The commercial anti-Nogo A antibody (lane 8) showed a 180-kDa immunoreactive band, which suggested that the target autoantigens of IgG from Cases 1, 2, 3, and 4 were different from Nogo-A. **(B)** WB analysis of extracted mouse sciatic nerve. Lane 1: IgG from Case 1 with anti-oligodendrocyte antibodies; lane 2: IgG from a HC. IgG from a representative MS patient with anti-oligodendrocyte antibodies (lane 1) or from a HC (lane 2) did not bind to specific immunoreactive bands. HC, healthy control; IgG, immunoglobulin G; MS, multiple sclerosis; RRMS, relapsing–remitting multiple sclerosis; WB, western blotting.

Western blotting (WB) of extracted mouse cerebellar proteins with IgG from anti-oligodendrocyte antibody-positive patients showed that IgG from one PPMS patient (Case 1 in Figure 1) specifically bound to protein bands of ~110 and 150 kDa, whereas IgG from two PPMS patients (Cases 2 and 3 in Figure 1) and one SPMS patient (Case 4 in Figure 1) or IgG from MS patients without anti-oligodendrocyte antibodies and HCs did not bind to specific bands (Figure 4A). The commercial anti-Nogo A antibody showed a 180-kDa immunoreactive band, which suggested that the target autoantigens of anti-oligodendrocyte antibodies were different from Nogo-A (Figure 4A). Moreover, IgG from Case 1 showed no specific immunoreactive bands against the sciatic nerve protein extracted from the peripheral nervous system (Figure 4B). These findings suggested that the target autoantigens of anti-oligodendrocyte antibodies from Case 1 might be ~110-kDa and/or 150-kDa proteins.

**Figure 5:**
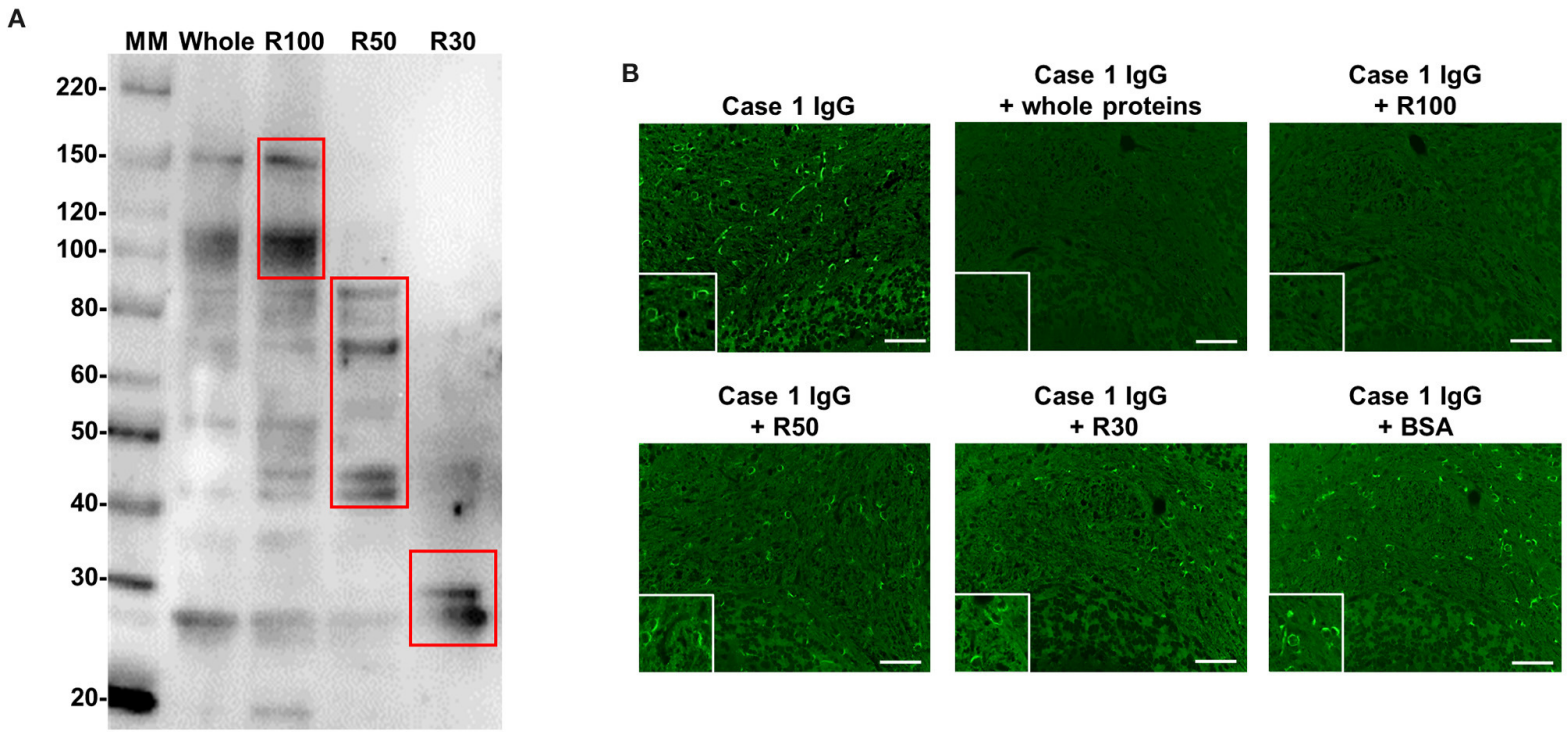
WB with IgG from Case 1 using whole protein and three different MW fractions (R100, R50, and R30) of mouse cerebellum and immunoadsorption assay by IFA. **(A)** WB with IgG from Case 1 in Figure 1 using whole protein and three different MW fractions (R100, R50, and R30) of mouse cerebellum. Whole protein and R100 fraction of mouse cerebellum included proteins of approximately 110 and 150 kDa, which were recognized by Case 1 IgG, whereas R50 and R30 fractions of mouse cerebellum did not include proteins with a MW larger than 100 kDa, which were recognized by Case 1 IgG. **(B)** Immunoadsorption assay by IFA. The immunostaining (green) of oligodendrocytes by IgG from Case 1 was removed by preincubation with whole protein and the R100 fraction of mouse cerebellum, whereas preincubation with R50, R30, or BSA did not remove the immunostaining (green) of oligodendrocytes by IgG from Case 1. Scale bar = 50 μm. IFA, immunofluorescence assay; IgG, immunoglobulin G; MM, molecular marker; MW, molecular weight; R, residue; WB, western blotting.

Next, we performed WB for three different MW fractions (R100, R50, and R30) of mouse cerebellum with IgG from Case 1. Protein bands of approximately 110 and 150 kDa were detected only in the R100 fraction as expected (Figure 5A). To confirm whether the proteins of ~110 kDa and/or 150 kDa included target autoantigens of anti-oligodendrocyte antibodies from Case 1, we performed immunoadsorption experiments with the R100 fraction by tissue-based IFA using mouse cerebellum and IgG from Case 1. We found that the staining of oligodendrocytes in Case 1 was removed by pre-incubation with the R100 fraction or whole extracted mouse cerebellum protein (Figure 5B). By contrast, pre-incubation with R50, R30, or BSA did not remove the staining of oligodendrocytes in Case 1 (Figure 5B). These experiments suggested that anti-oligodendrocyte antibodies from Case 1 recognized proteins with a MW larger than 100 kDa in mouse cerebellum, which might include proteins of approximately 110 kDa and/or 150 kDa. Next, we performed IP with purified IgG from Case 1 to identify the relevant autoantigen in protein bands of ~110 kDa and/or 150 kDa. However, we did not obtain specific IP samples.

### Characteristics of MS Patients With Anti-oligodendrocyte Antibodies

We compared the clinical features between 4 MS patients with anti-oligodendrocyte antibodies and 143 MS patients without anti-oligodendrocyte antibodies (Table 3). MS patients with anti-oligodendrocyte antibodies were significantly older at the time of serum sampling than MS patients without anti-oligodendrocyte antibodies (mean ± SD, years; 62.8 ± 5.7 vs. 45.1 ± 13.1, *p* = 0.0078). In addition, MS patients with anti-oligodendrocyte antibodies had significantly higher EDSS scores (mean ± SD; 6.63 ± 0.63 vs. 3.49 ± 2.50, *p* = 0.0214) and MSSS (7.34 ± 1.40 vs. 4.00 ± 2.95, *p* = 0.0361) than those without anti-oligodendrocyte antibodies, and a higher frequency of mental disturbance (75.0 vs. 19.6%, *p* = 0.0296) and CSF protein concentration (mean ± SD, mg/dl; 45.0 ± 4.5 vs. 35.5 ± 18.9, *p* = 0.0214).

**Table 3:** Demographic and clinical features in MS patients with and without anti-oligodendrocyte autoantibodies.

	**Anti-oligodendrocyte autoantibody-positive MS patients(*n* = 4)**	**Anti-oligodendrocyte autoantibody-positive MS patients(*n* = 143)**	***p-*value**
Female, *n* (%)	3/4 (75.0)	103/143 (72.0)	NS
Age at time of serum sampling (mean ± SD), years	62.8 ± 5.7	45.1 ± 13.1	0.0078
Age at disease onset (mean ± SD), years	41.3 ± 18.1	30.7 ± 11.4	NS
Disease duration at time of serum sampling (mean ± SD), years	21.5 ± 17.7	14.3 ± 11.0	NS
EDSS score at time of serum sampling (mean ± SD)	6.63 ± 0.63	3.49 ± 2.50	0.0214
MSSS at time of serum sampling (mean ± SD)	7.34 ± 1.40	4.00 ± 2.95	0.0361
Use of DMDs^a^, *n* (%)	0/4 (0.0)	63/143 (44.1)	NS
Neurological signs			
Pyramidal sign/motor weakness	4/4 (100.0)	108/143 (75.5)	NS
Visual impairment	0/4 (0.0)	24/143 (16.8)	NS
Cerebellar ataxia	3/4 (75.0)	54/143 (37.8)	NS
Sphincter disturbance	3/4 (75.0)	51/143 (35.7)	NS
Sensory impairment	3/4 (75.0)	76/143 (53.2)	NS
Mental disturbance	3/4 (75.0)	28/143 (19.6)	0.0296
Serum autoantibodies, *n* (%)			
cANA	0/4 (0.0)	12/143 (8.4)	NS
Anti-dsDNA antibodies	0/4 (0.0)	1/137 (0.7)	NS
Anti-SSA antibodies	1/4 (25.0)	4/137 (2.9)	NS
Anti-TPO antibodies	1/3 (33.3)	7/105 (6.7)	NS
CSF findings			
Cell count (/μl)	3.0 ± 2.2	4.9 ± 9.4	NS
Protein (mg/dl)	45.0 ± 4.5	35.5 ± 18.9	0.0384
Oligoclonal IgG bands, *n* (%)	1/3 (33.3)	63/100 (63.0)	NS
Increased IgG index^b^, *n* (%)	1/4 (25.0)	45/94 (47.9)	NS
CNS areas affected on MRI, *n* (%)			
Optic nerve	0/4 (0.0)	24/143 (16.8)	NS
Cerebral white matter	4/4 (100)	143/143 (100)	NS
Brainstem	2/4 (50)	81/143 (56.6)	NS
Cerebellum	1/4 (25.0)	57/143 (39.9)	NS
Spinal cord	3/4 (75.0)	118/143 (82.5)	NS

*The Mann–Whitney U-test was used to compare continuous variables, and the chi-square test or Fisher's exact probability test (when criteria for the chi-square test were not fulfilled) was used to compare categorical variables between patients with and without anti-oligodendrocyte antibodies*.

a *DMDs: interferon (IFN)β-1a, IFNβ-1b, glatiramer acetate, dimethyl fumarate, fingolimod*.

b *IgG index was considered increased if it was >0.658*.

*CNS, central nervous system; DMDs, disease-modifying drugs; dsDNA, double-stranded DNA; EDSS, Expanded Disability Status Scale; MRI, magnetic resonance imaging; MS, multiple sclerosis; MSSS, Multiple Sclerosis Severity Score; NS, not significant; RRMS, relapsing–remitting multiple sclerosis; SD, standard deviation; SPMS, secondary progressive multiple sclerosis; SSA, Sjögren's syndrome A; TPO, thyroid peroxidase*.

## Discussion

The main findings of the present study are as follows: (1) serum autoantibodies against the cytoplasm of oligodendrocytes in the CNS were detected in a small fraction of PPMS and SPMS patients, whereas RRMS, NMOSD, and OIND patients and HCs had no serum autoantibodies against oligodendrocytes; (2) the target CNS proteins recognized by serum anti-oligodendrocyte antibodies were ~110 kDa and/or 150 kDa; and (3) compared with anti-oligodendrocyte autoantibody-negative MS patients, MS patients with anti-oligodendrocyte antibodies were significantly older at the time of serum sampling, showing significantly greater disability and a higher frequency of mental disturbance.

The relationship between autoantibodies and CNS antigens has been an interesting issue in MS for many years (19). Regarding detection methods for autoantibodies, tissue-based IFA is a useful screening method to detect the presence of autoantibodies and their target cells in sera from patients with autoimmune neurological syndromes (20, 21). One previous tissue-based IFA study with unfixed frozen bovine brain sections and sera from MS patients showed that positive oligodendrocyte staining was present in 63% of MS patients, 43% of other neurological disease patients, and 29% of HCs (22). That study concluded that oligodendrocyte staining was not specific for MS and might be the result of non-specific binding to Fc receptors on oligodendrocytes. However, serum anti-CNS autoantibodies in MS were recently reinvestigated using tissue-based IFA with a fixation method, which instantly fixes vital tissues by whole-body perfusion with chilled paraformaldehyde (23). The fixation method improves tissue morphology and suppresses the ability of Fc receptors to bind to the Fc portion of IgG (23, 24). That study showed that positive oligodendrocyte staining was present in 5% (1/20) of NMOSD patients and 2.9% (4/136) of other neurological disease patients, whereas 0 of 106 MS patients had anti-oligodendrocyte antibodies although the types of MS (RRMS, SPMS, and PPMS) and clinical data were not described. Based on these findings, we screened anti-oligodendrocyte antibodies using tissue-based IFA with a similar fixation method and assessed the frequency of anti-oligodendrocyte antibodies in MS patients according to the types of MS. Using tissue-based IFA, we found that specific anti-oligodendrocyte antibodies were only present in a small fraction of progressive type MS patients with high MSSS and EDSS scores. Because all anti-oligodendrocyte antibodies in this study bound to the cytoplasm, but not the surface, of oligodendrocytes, this suggests they are induced by oligodendrocyte damage and are not involved in the initial pathogenic response (25). Given that anti-oligodendrocyte antibodies were not found in any patients with NMOSD or OIND despite CNS tissue damage, the production of anti-oligodendrocyte antibodies may be induced by oligodendrocyte disruption related to the neuroinflammation of progressive MS.

Our WB results using extracted mouse cerebellum proteins with IgG from anti-oligodendrocyte antibody-positive patients identified two candidate antigens of ~110 and 150 kDa in Case 1 by WB and immunoadsorption experiments. These antigens were not found by WB using sciatic nerves and IgG from Case 1, which is consistent with the results of IFA using peripheral nerves. Therefore, these anti-oligodendrocyte antibodies might reflect CNS-specific tissue damage. By contrast, IgG from Cases 2, 3, and 4 with anti-oligodendrocyte antibodies showed no significant immunoreactive bands in the WB of extracted mouse cerebellum proteins, probably because of the low titer of anti-oligodendrocyte antibodies or the autoreactivity of anti-oligodendrocyte antibodies against conformational epitopes. Because some autoantibodies showed a positive correlation with disease duration related to affinity maturation (26, 27), a reevaluation of WB with IgG from Cases 2, 3, and 4 is required in the future.

This study had several limitations. First, because of the low frequency of anti-oligodendrocyte antibodies, the sample size was not sufficient to obtain statistically significant results for clinical features. Second, unfortunately, we did not identify the target antigens of anti-oligodendrocyte antibodies by IP and mass-spectrometry-based identification methods. The future recruitment of a larger cohort of MS patients with anti-oligodendrocyte antibodies to identify target antigens is required. Tissue-based assays are generally of high specificity but low sensitivity. Therefore, once we have identified the target antigens of anti-oligodendrocyte antibodies, we will develop a more sensitive immunoassay, such as an enzyme-linked immunosorbent assay or a cell-based assay. This may lead to an increase in the positive rates of anti-oligodendrocyte antibodies and give further insight into the clinical significance of anti-oligodendrocyte antibodies in MS. Third, we used IFA and WB with mouse tissues for autoantibody screening and identification of the relevant autoantigen because the accurate evaluation of human antibodies using human tissue samples is generally difficult using indirect IFA because of co-detection of endogenous human immunoglobulins by secondary antibodies. Moreover, we unfortunately did not have appropriate human brain tissues for these experiments. When we perform IP with purified IgG and fresh human brain tissues in future studies, we may be able to obtain specific IP samples.

In conclusion, anti-oligodendrocyte autoantibodies were present in a small fraction of MS patients with progressive disease. Although the clinical significance of the anti-oligodendrocyte antibodies is still unclear because of their low frequency, they might be potential biomarkers to monitor disease progression in MS.

## Data Availability Statement

The raw data supporting the conclusions of this article will be made available by the authors, without undue reservation.

## Ethics Statement

The studies involving human participants were reviewed and approved by the Kyushu University Ethics Committee. The patients/participants provided their written informed consent to participate in this study. The animal study was reviewed and approved by the Institutional Animal Care and Use Committee at Kyushu University.

## Author Contributions

J-iK was the primary investigator of this study and was responsible for all the study processes, designed and conceptualized the study, interpreted the data, and revised the manuscript for intellectual content. YM designed and conceptualized the study, acquired the IFA and WB data, and analyzed the data. TF designed and conceptualized the study, analyzed the data, and drafted the manuscript for intellectual content. RY and DT analyzed and interpreted the data. KI acquired the WB data. AS, SF, TM, and NI contributed to the acquisition of clinical data from patients. KM and YN analyzed and interpreted the data. All authors contributed to the article and approved the submitted version.

## Conflict of Interest

The authors declare that the research was conducted in the absence of any commercial or financial relationships that could be construed as a potential conflict of interest. The handling editor declared a past co-authorship with one of the authors J-iK.

## Publisher's Note

All claims expressed in this article are solely those of the authors and do not necessarily represent those of their affiliated organizations, or those of the publisher, the editors and the reviewers. Any product that may be evaluated in this article, or claim that may be made by its manufacturer, is not guaranteed or endorsed by the publisher.
